# Dissecting stimulus-specific Ca^2+^ signals in amyloplasts and chloroplasts of *Arabidopsis thaliana* cell suspension cultures

**DOI:** 10.1093/jxb/erw038

**Published:** 2016-02-18

**Authors:** Simone Sello, Jennifer Perotto, Luca Carraretto, Ildikò Szabò, Ute C. Vothknecht, Lorella Navazio

**Affiliations:** ^1^Department of Biology, University of Padova, Via U. Bassi 58/B, 35131 Padova, Italy; ^2^Department of Biology I, Faculty of Biology, LMU Munich, Großhaderner Str. 2–4, D-82152 Munich, Germany

**Keywords:** Aequorin, amyloplasts, *Arabidopsis*, calcium signals, cell cultures, chloroplasts.

## Abstract

Calcium is used by plants as an intracellular messenger in the detection of and response to a plethora of environmental stimuli and contributes to a fine-tuned internal regulation. Interest in the role of different subcellular compartments in Ca^2+^ homeostasis and signalling has been growing in recent years. This work has evaluated the potential participation of non-green plastids and chloroplasts in the plant Ca^2+^ signalling network using heterotrophic and autotrophic cell suspension cultures from *Arabidopsis thaliana* plant lines stably expressing the bioluminescent Ca^2+^ reporter aequorin targeted to the plastid stroma. Our results indicate that both amyloplasts and chloroplasts are involved in transient Ca^2+^ increases in the plastid stroma induced by several environmental stimuli, suggesting that these two functional types of plastids are endowed with similar mechanisms for handling Ca^2+^. A comparison of the Ca^2+^ trace kinetics recorded in parallel in the plastid stroma, the surface of the outer membrane of the plastid envelope, and the cytosol indicated that plastids play an essential role in switching off different cytosolic Ca^2+^ signals. Interestingly, a transient stromal Ca^2+^ signal in response to the light-to-dark transition was observed in chloroplasts, but not amyloplasts. Moreover, significant differences in the amplitude of specific plastidial Ca^2+^ changes emerged when the photosynthetic metabolism of chloroplasts was reactivated by light. In summary, our work highlights differences between non-green plastids and chloroplasts in terms of Ca^2+^ dynamics in response to environmental stimuli.

## Highlight

*Arabidopsis* suspension-cultured cells stably expressing aequorin in the plastid stroma were used as a suitable tool to dissect differential Ca^2+^ responses of non-green plastids versus chloroplasts to several environmental cues.

## Introduction

In plants Ca^2+^ is used as an intracellular messenger to transduce a plethora of abiotic and biotic stimuli. A wide array of environmental stimuli have been shown to evoke specific intracellular spatio-temporal Ca^2+^ signals, which are further transduced by Ca^2+^ sensor proteins into transcriptional and metabolic responses (Dodd *et al.*, 2010; Whalley and Knight, 2013). Research on the intracellular compartmentalization of the ion has so far mainly focused on the vacuole, which is considered to be the major Ca^2+^ storage compartment in the plant cell, and for which extensive knowledge about Ca^2+^ transporters localized at the tonoplast is already available (Peiter, 2011; Martinoia *et al.*, 2012; Xu *et al.*, 2015). By contrast, the role of other intracellular compartments in orchestrating plant intracellular Ca^2+^ dynamics is still poorly understood, including that of chloroplasts, organelles peculiar to the plant cell. It has long been known that Ca^2+^ is involved in the modulation of photosynthesis (for a recent review, see e.g. Hochmal *et al.*, 2015), as well as in other plastidial processes, such as organelle division and the import of nuclear-encoded proteins (Kovács-Bogdán *et al.*, 2010; Rocha and Vothknecht, 2012; Stael *et al.*, 2012). Nevertheless, the role of Ca^2+^ in chloroplasts, and in plastids in general, is still elusive, with only little information so far available on the involvement of plastids in Ca^2+^ homeostasis and the generation of specific Ca^2+^ signals inside the plastids (Nomura and Shiina, 2014). Moreover, it has to be considered that different functional types of plastids are present in plants, and their structural and physiological differences may lead to differential involvement in the Ca^2+^ signalling network.

In an *in toto* experimental system, where root cells, stem cells, and leaf cells concurrently operate after signal perception to orchestrate an appropriate stimulus-specific response, non-green plastids and chloroplasts could act in distinct ways in the Ca^2+^-mediated transduction of signals. In order to better understand the involvement of different types of plastids in the Ca^2+^ signalling pathways of the plant cell, it is essential to monitor Ca^2+^ dynamics inside the organelle in a sensitive and accurate way, and to be able to safely discern the specific Ca^2+^ responses belonging to the different functional types of plastids (Jarvis and López-Juez, 2013). To allow us to dissect Ca^2+^ responses in non-green versus photosynthetic plastids, we generated cell suspension cultures containing either amyloplasts or chloroplasts from *Arabidopsis thaliana* seedlings stably expressing stroma-targeted aequorin (Mehlmer *et al.*, 2012). It has been shown that some stimulus-specific intracellular Ca^2+^ signals, sometimes limited to a specific tissue or cell type, may in fact be amplified in homogeneous, rapidly proliferating cell populations (Moscatiello *et al.*, 2013). The obtained data showed that different environmental cues triggered [Ca^2+^] elevations characterized by stimulus-specific kinetic parameters in the plastid stroma of both amyloplasts and chloroplasts, whereas the light-to-dark transition evoked a transient [Ca^2+^] change in the stroma of chloroplasts only. Moreover, significant differences in the amplitude of stromal [Ca^2+^] changes in response to specific abiotic stimuli were detected when dark-adapted chloroplasts were reactivated by light, suggesting that plastidial Ca^2+^ responses correlate with the photosynthetic status of the organelle.

## Materials and methods

### 

#### Chemicals

All chemicals, if not otherwise specified, were purchased from Sigma-Aldrich (St. Louis, MO, USA).

#### Plant material

*Arabidopsis thaliana* ecotype Columbia (Col-0) plant lines stably expressing the Ca^2+^-sensitive photoprotein aequorin fused to yellow fluorescent protein (YFP; designated ‘YA’) were used in this study. These transgenic plant lines have been genetically transformed with plasmids encoding YA chimera targeted to different intracellular locations: (i) the plastid stroma, by fusion with the first 85 amino acids of NADPH-dependent thioredoxin reductase C at the N-terminus of YA; (ii) the surface of the plastid outer envelope, by the fusion of the full-length outer envelope protein 7 (OEP7) at the N-terminus of YA; and (iii) the cytosol (excluding the nucleus), by the presence of the nuclear export signal from the heat-stable protein kinase inhibitor introduced between the cytosolic CPK17_G2A_ and YA. The whole expression cassettes of the different constructs were under the control of the cauliflower mosaic virus 35S promoter. All YA fusion vectors carried kanamycin resistance (Mehlmer *et al.*, 2012).

#### Set up of *A. thaliana* heterotrophic cell suspension cultures

Transgenic seeds were surface sterilized for 60s in a 70% ethanol, 0.05% Triton X-100 solution, then for 60s in 100% ethanol, and allowed to dry on an autoclaved Whatman paper disc for at least 10min. Seeds were subsequently plated on half-strength Murashige and Skoog (MS) medium containing 1.5% sucrose, 0.8% plant agar (Duchefa Biochemie, Haarlem, The Netherlands), and 50 µg/ml kanamycin. Cotyledons and hypocotyls from 7-day-old seedlings were transferred in axenic conditions onto callus induction medium (CIM) [Gamborg B5 basal medium, 0.5g/l MES, 2% sucrose, 0.8% agar, pH 5.7, 0.5mg/l 2,4-dichlorophenoxiacetic acid (2,4-D), and 0.05mg/l kinetin] supplemented with 50 µg/ml kanamycin, as described by Moscatiello *et al.* (2013). Callus fractions were transferred onto fresh CIM medium every 4 weeks two to three times, and then subcultured on MS medium containing 3% sucrose, 0.8% agar, pH 5.5, 0.5 µg/ml 2,4-D, 0.25 µg/ml 6-benzylaminopurine (BAP), and 50 µg/ml kanamycin. Initiation of cell suspension cultures was carried out by transferring portions of well-developed calli into liquid MS medium containing the same concentrations of sucrose and plant hormones as the solid medium, supplemented with 10 µg/ml kanamycin, and gently ground to obtain an adequate fragmentation of the calli. Cell suspension cultures were maintained at 24°C with a 16/8h light/dark photoperiod and an illumination of 25 µmol photons m^−2^ s^−1^ on a rotary shaker at 80rpm, and subcultured every week.

#### Set up of *A. thaliana* autotrophic cell suspension cultures

Autotrophic *A. thaliana* cell suspension cultures stably expressing stromal aequorin were initiated from heterotrophic cell suspension cultures by gradually decreasing the supply of organic carbon in the plant cell culture medium from 3% to 0.5% (w/w) sucrose to stimulate the photosynthetic activity. Moreover, the cultures were exposed to a relatively high illumination rate (110 µmol photons m^−2^ s^−1^) under an unvaried 16/8h light/dark cycle, to further stimulate the photosynthetic activity (Hampp *et al.*, 2012).

#### Microscopy observations

*A. thaliana* seedlings and calli were observed under a Leica MZ16 F fluorescence stereomicroscope. Images were acquired with a Leica DFC480 digital camera, using the Leica Application Suite (LAS) software. The intracellular localization of the YA chimeras in heterotrophic and autotrophic cell suspension cultures of *A. thaliana* was analysed using a Leica TCS SP5 II confocal laser scanning system mounted on a Leica DMI6000 inverted microscope. Excitation with the argon laser was carried out at 488nm and the emitted fluorescence was detected at 505–530nm for YFP and at 680–720nm for chlorophyll. Cells were also observed under a Leica DM5000 B microscope after staining with Lugol solution for starch granule detection. Images were acquired with a Leica DFC425 C digital camera, using the LAS software.

#### Pulse amplitude modulation analyses

Photosynthetic activity of autotrophic cell cultures was analysed in 6-day-old cultures by placing samples in a Closed FluorCam 800 MF (Photon Systems Instruments, Drasov, Czech Republic). Pulse amplitude modulation (PAM) fluorometric analysis expressed the photosynthetic efficiency as F_v_/F_m_, where F_v_ is the difference between the maximal (F_m_) and the basal (F_0_) fluorescence of chlorophyll. Chlorophyll fluorescence images were recorded using a CCD camera, and the data analysis was carried out using the FluorCam 7 software (Photon Systems Instruments).

#### Aequorin-based Ca^2+^ measurement assays

Mid-exponential phase (4-day-old) *A. thaliana* cell suspension cultures were reconstituted overnight with 5 µM coelenterazine (Prolume Ltd., Pinetop, AZ, USA) on a shaker at 80rpm, at 24°C in the dark. After extensive washing, a 50 µl cell aliquot was assayed using a luminometer (Electron Tubes Limited, Middlesex, UK) to monitor intracellular Ca^2+^ changes in response to selected environmental stimuli. Intracellular [Ca^2+^] dynamics were monitored after cell samples were treated with 10mM H_2_O_2_ (oxidative stress), 0.3M NaCl (salt stress), 0.6M mannitol (drought), three volumes of ice-cold cell culture medium (cold shock), and 20 µg/ml oligogalacturonides (OGs) with a degree of polymerization from 10 to 15 (pectic fragments of the plant cell wall that mimic a pathogen attack) (Moscatiello *et al.*, 2006). All stimuli, except cold shock, were prepared as 2-fold concentrated solutions in MS medium containing the appropriate concentration of sucrose for heterotrophic and autotrophic cell cultures, respectively. In addition, the effect of the light-to-dark transition on stromal [Ca^2+^] in amyloplasts and chloroplasts was tested by transferring suspension-cultured cells into the luminometer chamber at the end of the 16h light cycle and measuring dark-induced [Ca^2+^] changes. Ca^2+^ responses to the previously mentioned stimuli were also monitored after 16h light reactivation of the photosynthetic metabolism of chloroplasts. At the end of each experiment the remaining aequorin pool was discharged by injecting 0.33M CaCl_2_ and 10% ethanol into the cell samples. The light signal was collected and converted off-line into [Ca^2+^] values using a computer algorithm based on the Ca^2+^ response curve of aequorin (Brini *et al.*, 1995).

#### Statistical analysis

Data are shown as means ± SE of at least three independent experiments, and the differences between groups were assessed by Student’s *t*-test.

## Results

### Ca^2+^ measurement assays in *A. thaliana* heterotrophic cell cultures revealed the participation of amyloplasts in Ca^2+^ handling and in the generation of stromal Ca^2+^ signals

**Fig. 1.**
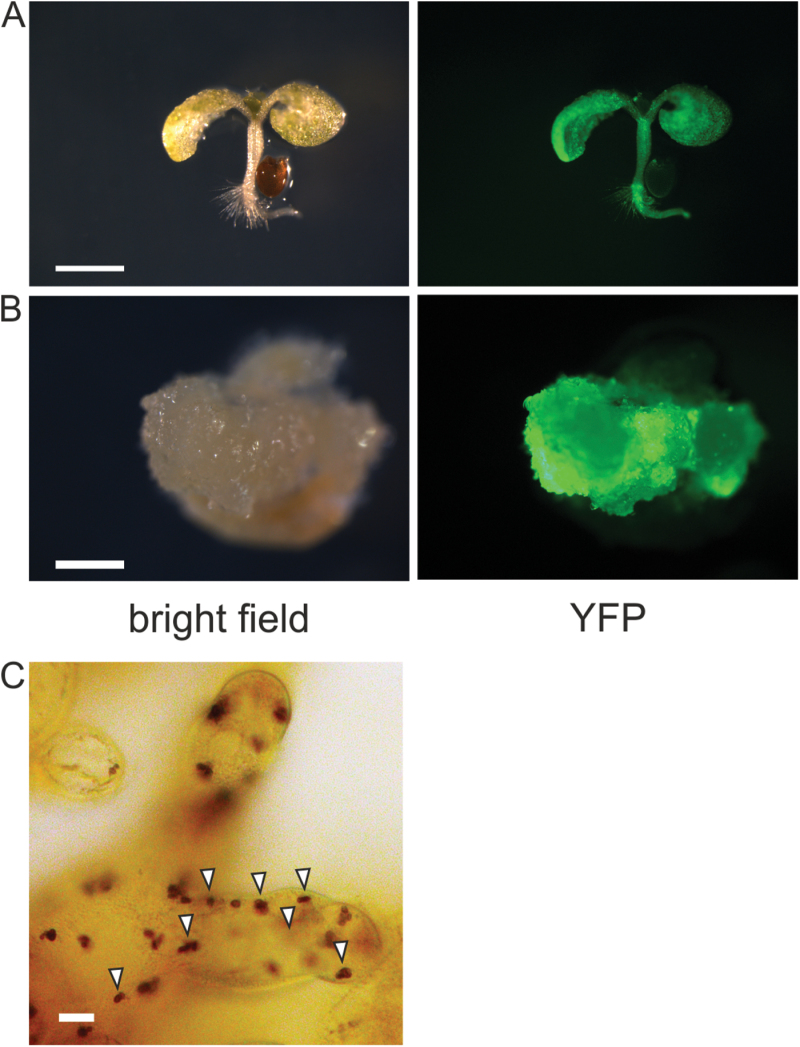
Set up of *Arabidopsis thaliana* heterotrophic cell cultures stably expressing the Ca^2+^-sensitive photoprotein aequorin in the plastid stroma. Explants from seedlings expressing the YA chimera and showing a good level of fluorescence (**A**) were placed on CIM to make hypocotyls and cotyledons dedifferentiate. After ~1 month explants produced well-developed calli (**B**) that were subsequently transferred onto solid MS medium, and then to liquid MS medium containing 3% sucrose and the appropriate concentrations of phytohormones to obtain rapidly proliferating cell suspension cultures. The same method was applied to set up heterotrophic cell cultures stably expressing aequorin in the plastid outer envelope and cytosol. A, B: bar, 1mm. (**C**) Staining of suspension-cultured cells with Lugol solution. Starch granules inside amyloplasts are indicated by white arrowheads. Bar, 10 µm. This figure is available in colour at *JXB* online.

**Fig. 2.**
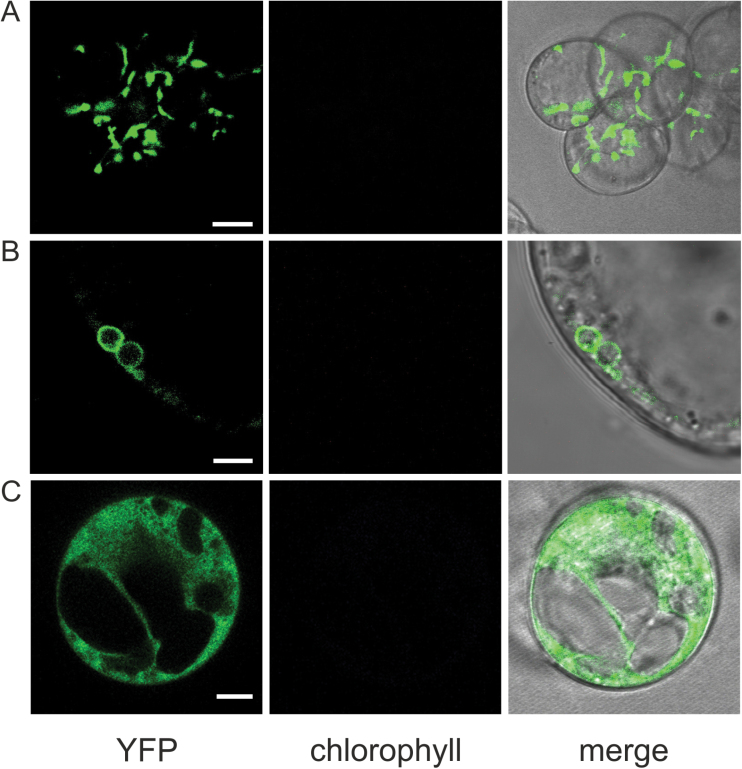
Confocal microscopy analysis of YA localization in *A. thaliana* suspension-cultured cells stably expressing the different chimeras in the plastid stroma (**A**), outer envelope (**B**), and cytosol (**C**). Bars: 10 µm (A, C), 5 µm (B). This figure is available in colour at *JXB* online.

Plant cell cultures are widely used as a useful and versatile experimental system to dissect the complexity of the plant organism *in toto*, permitting the analysis of a wide array of physiological processes at the cellular level. In particular, suspension-cultured cells are a valuable tool to analyse many different Ca^2+^-based signal transduction pathways (Navazio *et al.*, 2007a, b; Lachaud *et al.*, 2010; Amelot *et al.*, 2011; Manzoor *et al.*, 2012). We obtained *in vitro* cultures after the dedifferentiation of cotyledons and hypocotyls from *A. thaliana* lines stably expressing the bioluminescent Ca^2+^ reporter aequorin in the plastid stroma, the cytosolic surface of the outer membrane of the plastid envelope, and the cytosol, respectively. All constructs used for plant transformation carried a YA fusion (Mehlmer *et al.*, 2012) that enabled an easier screening of the most promising seedlings to generate *in vitro* cultures, based on YFP fluorescence (Fig. 1A). About 1 month after the transfer of axenic explants onto dedifferentiating medium (CIM), fluorescent calli were formed (Fig. 1B); they were subsequently transferred into liquid medium (MS medium containing 3% sucrose, supplemented with 0.5 µg/ml 2,4-D and 0.25 µg/ml BAP) to get cell suspension cultures (see Supplementary Fig. S1 at *JXB* online). Lugol staining highlighted starch granules inside the plastids (Fig. 1C), which, together with the lack of chlorophyll fluorescence (Fig. 2, middle column), indicated that the cell suspension cultures contained amyloplasts as their functional type of plastids. Confocal microscopy analyses confirmed the correct intracellular localizations of all the YA chimeras, which were targeted to the plastid stroma (Fig. 2A), the outer membrane of the plastid envelope (Fig. 2B), and the cytosol (Fig. 2C), respectively.

**Fig. 3.**
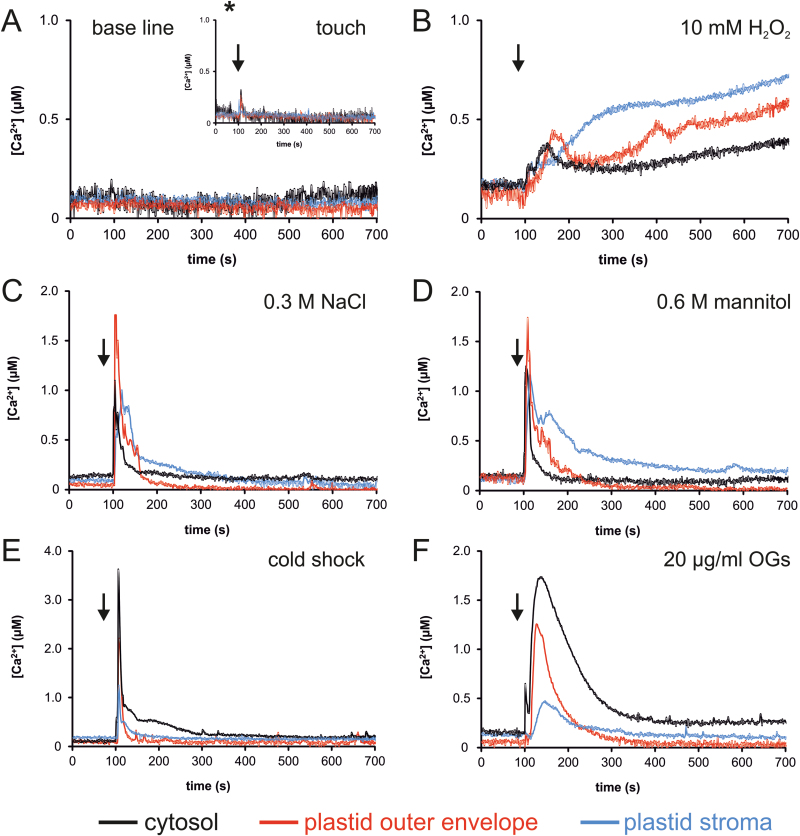
Monitoring of intracellular Ca^2+^ dynamics in response to different environmental stimuli in *A. thaliana* heterotrophic cell suspension cultures containing amyloplasts and stably expressing aequorin in the cytosol (black trace), plastid outer envelope (red trace), and stroma (blue trace). Ca^2+^ measurement assays were carried out in resting conditions (**A**) and in response to abiotic (**B–E**) and biotic (**F**) stimuli, as indicated in each panel of the figure. In the insert (asterisk) the effect of the injection of an equal volume of cell culture medium on [Ca^2+^] is reported as a control for stimulus administration. Cells were challenged with the different stimuli after 100s (arrow). These and the following Ca^2+^ traces are representative of at least three independent experiments giving similar results.

Heterotrophic cell cultures were challenged with different environmental stimuli with well-established Ca^2+^-mediated signal transduction. This allowed us to monitor in parallel Ca^2+^ dynamics in the bulk cytosol, the cytosolic microdomain just outside plastids (by means of the aequorin chimera targeted to the cytosolic surface of the plastid outer envelope), and the organelle stroma. [Ca^2+^] in all these subcellular locations was between 100 and 200nM in resting conditions, indicating a similarly low Ca^2+^ level in both cytosol and plastid stroma (Fig. 3A). Upon treatment with different abiotic and biotic stimuli, transient increases in [Ca^2+^] were recorded in all subcellular localizations, characterized by different kinetic parameters, as illustrated in the shown representative experiments (Fig. 3B–F). Because all chemicals were dissolved in cell culture medium, cells were challenged with an equal volume of iso-osmotic cell culture medium as a touch control (mechanical perturbation), which induced a rapid and small increase in [Ca^2+^] that quickly dissipated (Fig. 3A, insert).

In response to oxidative stress (10mM H_2_O_2_, Fig. 3B) a cytosolic increase in [Ca^2+^] was recorded, peaking at 0.24±0.06 µM after 49±5s (n = 5). In the cytosolic microdomain close to the surface of the amyloplasts (outer envelope), the maximum [Ca^2+^] value observed was recorded at 0.35±0.02 µM after 54±4s (n = 3). In the amyloplast stroma the oxidative stress induced a slower increase in [Ca^2+^] that did not reach a plateau but continued to increase in the considered time interval. Interestingly, the ascending stromal Ca^2+^ trace matched the decrease in cytosolic [Ca^2+^] level, suggesting a potential involvement of plastids in the dissipation of the Ca^2+^ transient in the cytosol.

Cell treatment with salt stress (0.3M NaCl, Fig. 3C) or drought stress (0.6M mannitol, Fig. 3D) induced rapid increases in [Ca^2+^] in all the considered sub-compartments. In response to both stimuli, transient microdomains of high [Ca^2+^] were recorded close to the organelle outer envelope, characterized by a higher amplitude [1.26±0.16 µM with 0.3M NaCl (n = 5) and 1.37±0.21 µM with 0.6M mannitol (n=6)] than that of the corresponding [Ca^2+^] changes in the bulk cytosol [0.66±0.11 µM with 0.3M NaCl (n = 5) and 0.92±0.11 µM with 0.6M mannitol (n = 8)]. Interestingly, the decrease in [Ca^2+^] in both the bulk cytosol and cytosolic microdomain at the plastid surface temporally coincided with the increase of [Ca^2+^] in the stroma [peaking at 0.79±0.08 µM for 0.3M NaCl (n = 6) and 0.92±0.11 µM for 0.6M mannitol (n = 8)], again suggesting a role for non-green plastids as Ca^2+^ sinks involved in the modulation of intracellular Ca^2+^ signals.

Cold shock, simulated by injection of three volumes of ice-cold cell culture medium, resulted in an immediate [Ca^2+^] elevation in all three subcellular localizations, characterized by different magnitudes (Fig. 3E). The highest increase (3.45±0.19 µM at 6±1s, n = 4) was recorded in the cytosol, whereas the [Ca^2+^] peaks monitored at the plastid surface (1.61±0.29 µM, n = 5) and stroma (0.79±0.13 µM, n = 4) were progressively lower, although characterized by equally fast kinetics. Indeed it is known that cold shock activates a rapid [Ca^2+^] increase in the cytosol due to the influx of Ca^2+^ from the extracellular medium and a release of the ion from the vacuole (Knight *et al.*, 1996).

OGs with a degree of polymerization from 10 to 15 (i.e. pectic fragments of the plant cell wall that mimic a pathogen attack; Moscatiello *et al.*, 2006) were used as a biotic stimulus (Fig. 3F). OGs (20 µg/ml) induced a remarkable increase in [Ca^2+^] in the cytosol that peaked at 1.67±0.08 µM after 27±5s (n = 3) and lasted for ~5min. A similar response was observed at the outer membrane surface subdomain, albeit with a slightly lower amplitude (1.00±0.19 µM, n = 4). OGs were even found to evoke a transient Ca^2+^ increase in the plastid stroma, characterized by similar kinetics but a lower amplitude (0.39±0.04 µM, n = 7). Taken together, the data obtained indicate that in resting conditions Ca^2+^ is kept at a similar concentration in the cytosol and in the stroma of amyloplasts in *A. thaliana* suspension-cultured cells, and that various environmental stresses evoke differential stromal [Ca^2+^] signals, specific for each stimulus. Moreover, analysis of the kinetics of the stromal Ca^2+^ transients demonstrated that, at least in some cases (oxidative stress, salinity, drought), plastids seem to be involved in the dissipation, rather than the generation, of the stimulus-induced cytosolic Ca^2+^ increases.

### Differential Ca^2+^ signals are recorded between chloroplasts and amyloplasts when the photosynthetic metabolism of autotrophic cell cultures is reactivated by light

**Fig. 4.**
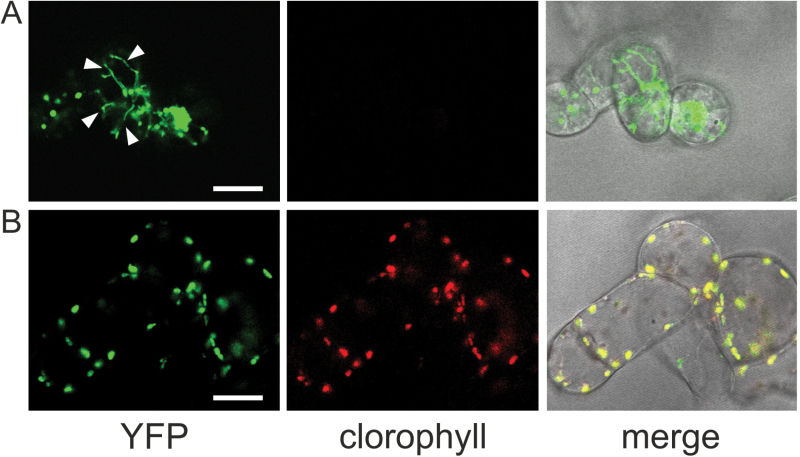
Set up of *A. thaliana* photoautotrophic cell suspension cultures stably expressing YA in the stroma of chloroplasts (**B**), starting from the corresponding heterotrophic cell cultures (**A**). Confocal microscopy analysis demonstrated the presence of much more evident stromules (white arrowheads) in the amyloplasts of heterotrophic cell cultures (A) than in the chloroplasts of autotrophic cell cultures (B). Bar, 20 µm. This figure is available in colour at *JXB* online.

In order to compare stromal Ca^2+^ dynamics between amyloplasts and chloroplasts, we prepared *Arabidopsis* photoautotrophic cell cultures expressing aequorin in the plastid stroma (see ‘Materials and methods’ and Supplementary Fig. S1 at *JXB* online). Confocal microscopy analysis showed that in the autotrophic cell line, unlike in the corresponding heterotrophic line, plastids displayed a remarkable level of chlorophyll autofluorescence, confirming that the plastids present were chloroplasts (Fig. 4B). It should be noted that stromules, dynamic stroma-filled tubules continuously extending and retracting from plastids, were much more evident in amyloplasts than chloroplasts from *Arabidopsis* suspension-cultured cells (Fig. 4 and Supplementary Videos S1 and S2). PAM assays demonstrated the high photosynthetic efficiency of the autotrophic cell cultures (F_v_/F_m_ = 0.75±0.03, n = 3; Supplementary Fig. S1). It was not possible to calculate the F_v_/F_m_ parameter in the heterotrophic cell cultures because they contain amyloplasts, which do not possess thylakoids and therefore lack chlorophyll and photosynthetic activity.

**Fig. 5.**
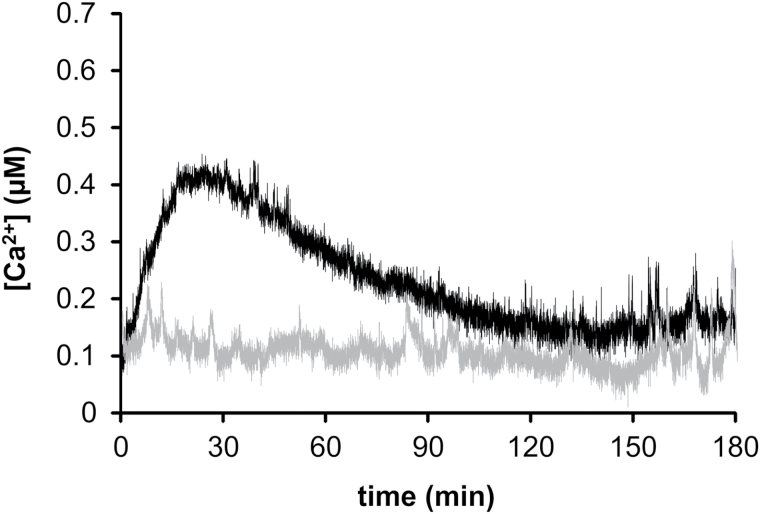
Effect of light-to-dark transition on plastid stromal [Ca^2+^] in *A. thaliana* autotrophic and heterotrophic cell suspension cultures. A dark-induced Ca^2+^ transient was observed in the stroma of chloroplasts (black trace) but not amyloplasts (grey trace).

To analyse the potentially different involvement of amyloplasts and chloroplasts in Ca^2+^ homeostasis and signalling, autotrophic cell cultures stably expressing stromal aequorin were challenged with the same stimuli described above for heterotrophic cell cultures. The results did not highlight significant differences in the Ca^2+^ dynamics recorded in the stroma of non-green versus green plastids, concerning either the amplitude or the timing of the maximal Ca^2+^ peak (Supplementary Figs S2 and S3). However, a clear difference in their Ca^2+^ response emerged when a stimulus more closely related to the specific physiology of the chloroplast was tested, that is, the light-to-dark transition at the end of the 16h light photoperiod. In this latter case, a transient [Ca^2+^] elevation was detected in the stroma of chloroplasts, peaking at ∼0.44 µM about 20min after light switch-off and falling back to basal values within 2h (Fig. 5, black trace). By contrast, no changes in [Ca^2+^] were observed in the stroma of amyloplasts (Fig. 5, grey trace).

**Fig. 6.**
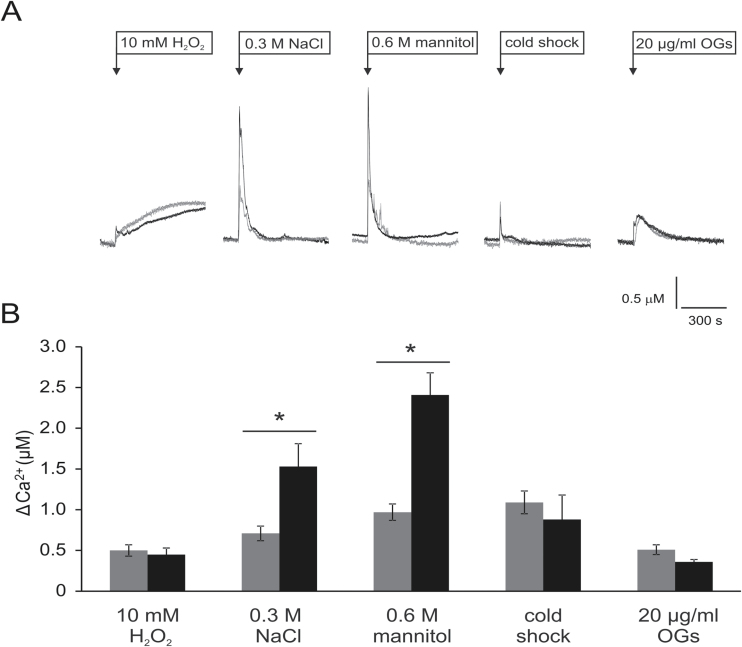
Representative Ca^2+^ traces (**A**) and the means of the maximum [Ca^2+^] increase over basal [Ca^2+^] (ΔCa^2+^]) (**B**) to compare Ca^2+^ dynamics in *A. thaliana* autotrophic cell suspension cultures expressing aequorin in the chloroplast stroma, after exposure to 8h dark (A, grey traces; B, grey columns) or 16h light to reactivate the photosynthetic metabolism (A, black traces; B, black columns). Suspension-cultured cells were challenged with the environmental stimuli indicated at the bottom of the columns. Statistically significant differences (*P* < 0.05) in the Δ[Ca^2+^] are indicated by an asterisk.

To determine whether chloroplasts display differential Ca^2+^ responses to other stimuli as a function of their metabolic state, Ca^2+^ dynamics were compared between autotrophic cell cultures directly after exposure to 8h dark and after reactivation of photosynthesis by exposure to 16h light. Light exposure did not alter the overall stromal Ca^2+^ response to either oxidative stress, cold shock, or OGs (Fig. 6). However, a significantly higher increase in [Ca^2+^] was recorded in the stroma of light-reactivated chloroplasts than in non-reactivated chloroplasts in response to salt stress (light-reactivated, 1.53±0.28 µM, n = 3; non-reactivated, 0.71±0.09 µM, n = 4) and drought (light-reactivated, 2.41±0.27 µM, n = 4; non-reactivated, 0.97±0.10 µM, n = 4). These results indicate that, although green plastids displayed Ca^2+^ responses that were indistinguishable from those of non-green plastids when their photosynthetic metabolism was switched off by dark exposure (Supplementary Figs S2 and S3), clear differences emerged after their photosynthetically active status was reactivated by 16h light exposure (Fig. 6). The effective reactivation of photosynthesis, leading to starch biosynthesis after CO_2_ assimilation into photosynthates, was confirmed by the presence of Lugol-stained starch granules in the stroma of autotrophic suspension-cultured cells after 16h light, but not after 8h dark (Supplementary Fig. S4).

## Discussion

To dissect the involvement of chloroplasts and non-green plastids in Ca^2+^ handling during signal transduction events, we monitored changes in [Ca^2+^] in response to different environmental stimuli in *A. thaliana* heterotrophic and autotrophic cell suspension cultures stably expressing aequorin targeted to the organelle stroma. The results presented in this work provide evidence that amyloplasts, that is, non-green plastids specialized in the accumulation of starch, have tightly regulated [Ca^2+^] inside the organelle and the ability to evoke specific Ca^2+^ changes in response to several environmental cues. It is known that Ca^2+^ plays essential regulatory roles in the physiology of plastids by affecting diverse processes that do not necessarily relate only to photosynthesis, such as plastid division and the import of nucleus-encoded proteins (Rocha and Vothknecht, 2012; Stael *et al.*, 2012; Nomura and Shiina, 2014; Hochmal *et al.*, 2015). Nevertheless, a comparison between non-green plastids and chloroplasts in terms of Ca^2+^ dynamics in response to environmental stimuli had never previously been addressed. On the basis of the peculiar structural and physiological features of the different functional types of plastids (Jarvis and López-Juez, 2013), distinct responses in terms of Ca^2+^ homeostasis and signalling might be expected. Surprisingly, no significant differences were detected in terms of stromal Ca^2+^ dynamics when comparing heterotrophic and dark-adapted autotrophic suspension-cultured cells in response to the considered stimuli. The absence of distinct Ca^2+^ signatures in non-green versus green plastids suggests that the two functional types of plastids possess similar mechanisms that allow for Ca^2+^ flux in plastids. In resting conditions, the cytosol and the amyloplast stroma had very similar [Ca^2+^]. In response to salt, drought, and oxidative stresses, the timing of the Ca^2+^ peaks recorded in the different subcellular locations (i.e. the bulk cytosol, the plastid outer envelope, and the stroma) were gradually delayed from the cytosol moving towards the plastid stroma, indicating a likely crucial role for plastids in shaping and switching off intracellular Ca^2+^ signals. The kinetics of the observed Ca^2+^ changes—that is, the fact that a decrease in [Ca^2+^] at the cytosolic surface of the outer membrane of the envelope often correlated with an increase of [Ca^2+^] in the stroma—support the notion that under these conditions plastids may function as Ca^2+^ sinks, used by the cell to dissipate cytosolic [Ca^2+^] signals, thus contributing to the modulation of intracellular Ca^2+^ signatures.

In view of the importance of the light stimulus for the physiology of plastids, we evaluated the effect of the light-to-dark transition on stromal Ca^2+^ levels in chloroplasts and non-green plastids. Previous studies have demonstrated that a long-lasting transient [Ca^2+^] elevation is detected in the chloroplast stroma after a lights-off stimulus (Sai and Johnson, 2002) and further investigations proved that this elevation is restricted to the organelle and does not involve the cytosol (Nomura *et al.*, 2012). Our aequorin-based Ca^2+^ measurement experiments, carried out in heterotrophic and autotrophic suspension-cultured cells containing amyloplasts or chloroplasts, respectively, allowed us to examine the potential differential response of these two functional types of plastids to the light-to-dark transition. Our data showed that the dark-induced Ca^2+^ transient occurred in green plastids only, suggesting a potential role of the thylakoid system (exclusively present in chloroplasts) in generating the stromal Ca^2+^ signal. A likely possibility is that the observed stromal Ca^2+^ fluxes derive from the thylakoid lumen, which may act as a mobilizable Ca^2+^ store. Alternatively, complexed Ca^2+^ may be released from the thylakoid membrane, without the involvement of Ca^2+^ flux-permitting transporters localized at the thylakoid membrane. Indeed, there is a plethora of Ca^2+^-binding proteins in the chloroplast (Rocha and Vothknecht, 2012; Stael *et al.*, 2012) and upon the light-to-dark transition these proteins may release Ca^2+^ into the surrounding environment. Moreover, the calcium-sensing receptor CAS, a low-affinity, high-capacity Ca^2+^-binding protein localized in thylakoid membranes, may be a major actor in this process (Nomura *et al.*, 2008; Vainonen *et al.*, 2008; Weinl *et al.*, 2008). Indeed, the dark-induced stromal Ca^2+^ transient was found to be markedly reduced in a *CAS* knockout background (Nomura *et al.*, 2012). Accurate estimates of [Ca^2+^] and its changes inside and around the thylakoid membrane system are urgently needed to shed light on the origin of these intra-plastidal Ca^2+^ fluxes.

The finding of a stromal Ca^2+^ increase occurring shortly after the light-to-dark transition and restricted to chloroplasts prompted us to more closely analyse the potential light dependency of chloroplast Ca^2+^ responses to environmental cues. Autotrophic cell suspension cultures were exposed to a 16h light period to allow for the reactivation of the photosynthetic metabolism, and stromal Ca^2+^ dynamics of dark-adapted and light-reactivated cells were compared after the perception of oxidative stress, salinity, drought, cold shock, and a mimicked pathogen attack (treatment with OGs). Interestingly, significant differences were observed upon challenge with either of the two hyperosmotic shocks simulating salinity (0.3M NaCl) and drought (0.6M mannitol), whereby more than a 2-fold higher increase in [Ca^2+^] was detected in light-reactivated chloroplasts. The remarkable changes in the amplitude of the stromal Ca^2+^ responses between light-reactivated and dark-adapted chloroplasts, but not between amyloplasts and dark-adapted chloroplasts, infer an indirect impact of light on the Ca^2+^-mediated transduction mechanisms of these stresses. Indeed, it has been demonstrated that drought and salinity alter the normal homeostasis of cells and cause an increased production of reactive oxygen species (ROS) that can be used by the plant as signalling molecules in retrograde signalling (Miller *et al.*, 2010). Because ROS-mediated retrograde signalling requires ROS produced by chloroplasts during the light phase, one may speculate that Ca^2+^-mediated responses to environmental stimuli might differ in light-reactivated green plastids compared to dark-adapted ones. The relevance of interconnected networks of Ca^2+^ and ROS signals is increasingly emerging in plant systemic signalling (Gilroy *et al.*, 2014).

In conclusion, this work shows that *A. thaliana* cell suspension cultures stably expressing the bioluminescent Ca^2+^ reporter aequorin targeted to the plastid stroma can be used as a versatile experimental tool to analyse potential differences in Ca^2+^ dynamics between chloroplasts and non-green plastids during signal transduction events. The detection of delayed transient stromal Ca^2+^ elevations, often temporally corresponding with a decline in the magnitude of cytosolic [Ca^2+^] changes, suggests that plastids function as Ca^2+^ stores, involved in switching off the increase of cytosolic Ca^2+^, and thus in shaping intracellular Ca^2+^ signals. A more direct role for these intra-plastidial Ca^2+^ fluxes, and for the consequent changes in [Ca^2+^], in the specific physiology of the organelle, both chloroplasts and amyloplasts, cannot be ruled out and needs to be further investigated. Chloroplast-mediated activation of plant immune signalling, via specific Ca^2+^ signals evoked in the chloroplast stroma by the pathogen-associated molecular pattern flg22 has already been demonstrated in *A. thaliana* (Nomura *et al.*, 2012). Chloroplast Ca^2+^ signatures were also recorded in response to other plant defence elicitors, such as cryptogein and OGs (Manzoor *et al.*, 2012). In this paper the OG-induced stromal Ca^2+^ signal was not restricted only to chloroplasts, but also occurred in amyloplasts, suggesting a potential role for this functional type of plastid, most abundant in the root, in Ca^2+^-mediated signalling during pathogenic as well as beneficial plant–microbe interactions (Moscatiello *et al.*, 2012).

Future work should address the characterization of different Ca^2+^ transporters localized at plastidial membranes, which have just recently started to be identified (Teardo *et al.*, 2010, 2011, 2015; Hochmal *et al.*, 2015). Moreover, the involvement of stromules as well as additional direct contact sites between plastids and other intracellular compartments, such as the endoplasmic reticulum (Schattat *et al.*, 2011; Mehrshahi *et al.*, 2013; Brunkard *et al.*, 2015), needs to be elucidated in the context of the potential bidirectional exchange of Ca^2+^ during signalling events.

## Supplementary data

Supplementary data are available at *JXB* online.

Supplementary Fig. S1. *A. thaliana* heterotrophic (A) and autotrophic (B) cell suspension cultures stably expressing aequorin in the stroma. (C) PAM imaging analysis of autotrophic cell suspension cultures of *A. thaliana* (F_v_/F_m_ value = 0.79).

Supplementary Fig. S2. Comparison between stromal Ca^2+^ dynamics in amyloplasts and dark-adapted chloroplasts after the perception of environmental stimuli.

Supplementary Fig. S3. Analysis of stromal Ca^2+^ dynamics in terms of the difference between peak [Ca^2+^] and basal [Ca^2+^] (Δ[Ca^2+^]) and of the timing of the [Ca^2+^] peak after stimulus injection (Δt) in *A. thaliana* heterotrophic (grey columns) and dark-adapted autotrophic (black columns) cell suspension cultures in response to different environmental stimuli.

Supplementary Fig. S4. Reactivation of photosynthetic metabolism by 16h light exposure in autotrophic *A. thaliana* cell cultures.

Supplementary Video S1. Single-cell z-stack series of *A. thaliana* heterotrophic cell suspension cultures stably expressing YA targeted to the plastid stroma. Stromules, dynamic stroma-filled tubules extending from amyloplasts, are evident.

Supplementary Video S2. Single-cell z-stack series of autotrophic *A. thaliana* cell suspension cultures stably expressing YA targeted to the chloroplast stroma. The yellow signal indicates co-localization of YFP fluorescence and chlorophyll autofluorescence.

Supplementary Data
