# Supplementary material for: Dissecting stimulus-specific Ca2+ signals in amyloplasts and chloroplasts of Arabidopsis thaliana cell suspension cultures
Source: J Exp Bot. 2016 Feb 18;67(13):3965–74. doi: 10.1093/jxb/erw038 (PMC4915524; doi:10.1093/jxb/erw038)
Supplement: Supplementary Data [file supp_67_13_3965__index.html]

Dissecting stimulus-specific Ca2+ signals in amyloplasts and chloroplasts of Arabidopsis thaliana cell suspension cultures — Dissecting stimulus-specific Ca2+ signals in amyloplasts and chloroplasts of Arabidopsis thaliana cell suspension cultures — Supplementary Data 

# Dissecting stimulus-specific Ca2+ signals in amyloplasts and chloroplasts of *Arabidopsis thaliana* cell suspension cultures

## Supplementary Data

Data files

- SupplementaryFig.1\_4.pdf - Supplementary Data
- jexbot166389\_file002.mov - Supplementary Data
- jexbot166389\_file003.mov - Supplementary Data
