# Supplementary material for: Dissecting stimulus-specific Ca2+ signals in amyloplasts and chloroplasts of Arabidopsis thaliana cell suspension cultures
Source: J Exp Bot. 2016 Feb 18;67(13):3965–74. doi: 10.1093/jxb/erw038 (PMC4915524; doi:10.1093/jxb/erw038)
Supplement: Supplementary Data [file supp_erw038_SupplementaryFig.1_4.pdf]

Journal of Experimental Botany

Dissecting stimulus-specific Ca^{2+} signals in amyloplasts and chloroplasts of *Arabidopsis thaliana* cell suspension cultures

Simone Sello, Jennifer Perotto, Luca Carraretto, Ildikò Szabò, Ute C. Vothknecht, Lorella Navazio

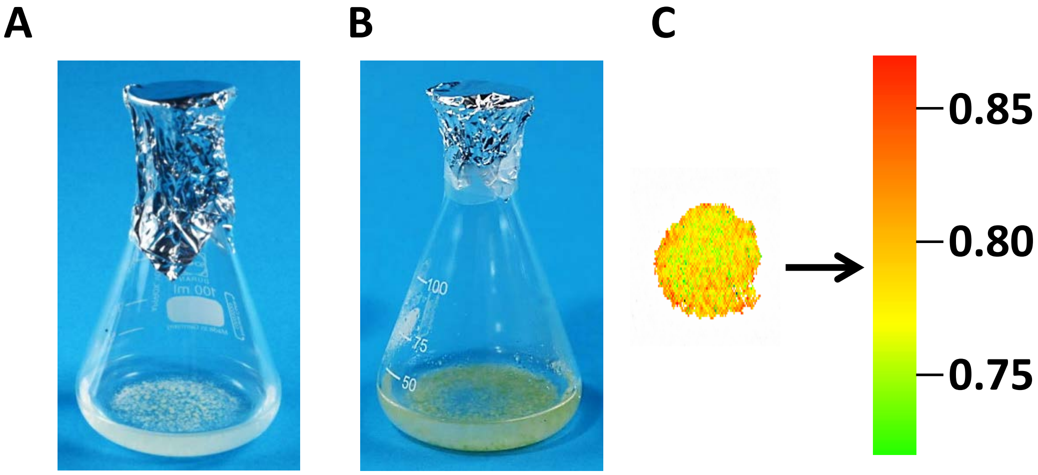

Supplementary Fig. S1. *A. thaliana* heterotrophic (A) and autotrophic (B) cell suspension cultures stably expressing aequorin in the stroma. C) PAM imaging analysis of *A. thaliana* autotrophic cell suspension cultures (F_v/F_m value = 0.79).

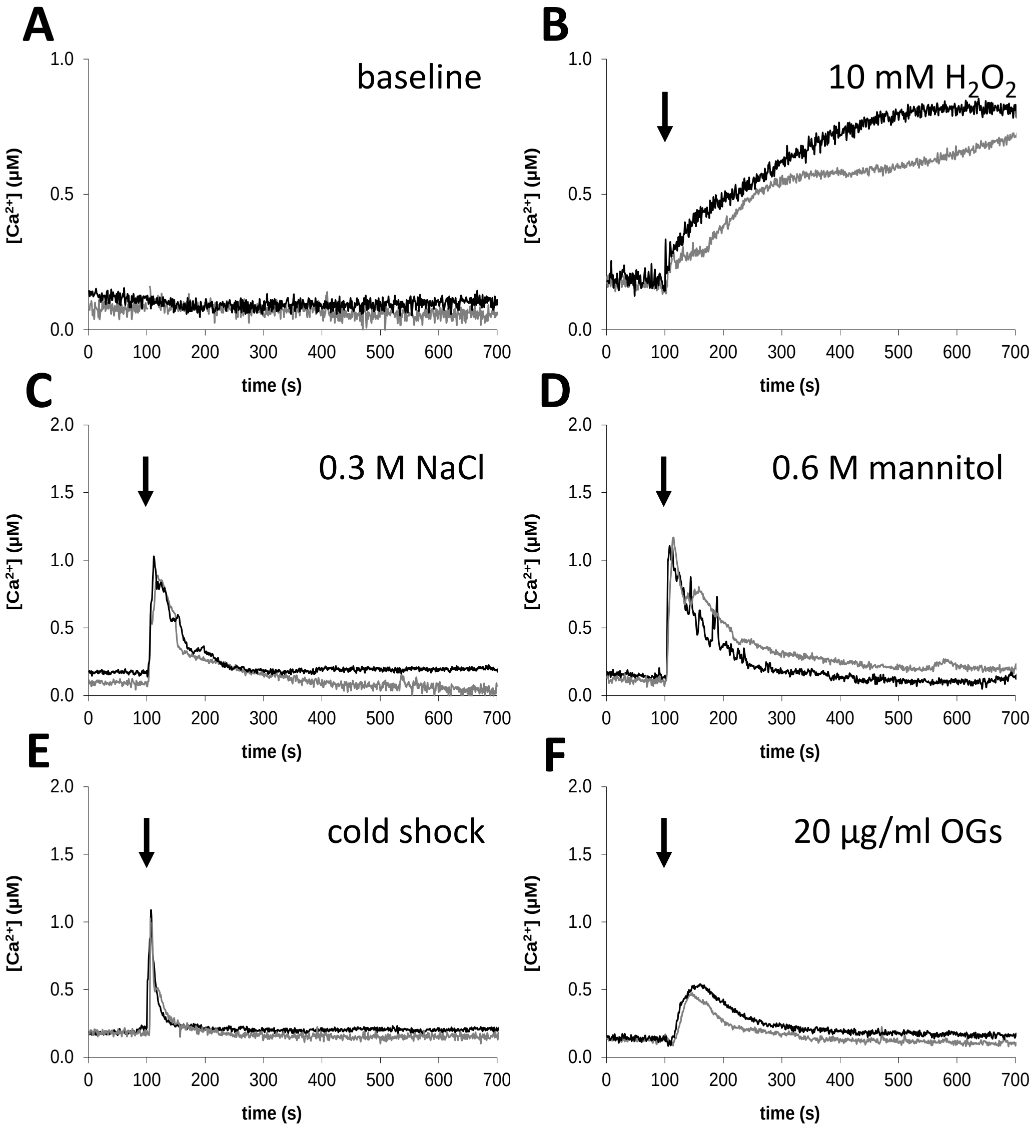

Supplementary Fig. S2. Comparison between stromal Ca^{2+} dynamics in amyloplasts and dark-adapted chloroplasts after the perception of environmental stimuli. *A. thaliana* heterotrophic (grey trace) and autotrophic (black trace) cell suspension cultures containing amyloplasts and chloroplasts, respectively, and stably expressing stroma-targeted aequorin were challenged (after 100 s, arrow) with different abiotic and biotic stimuli as specified in each panel. Ca^{2+} traces are representative of at least three independent experiments giving similar results.

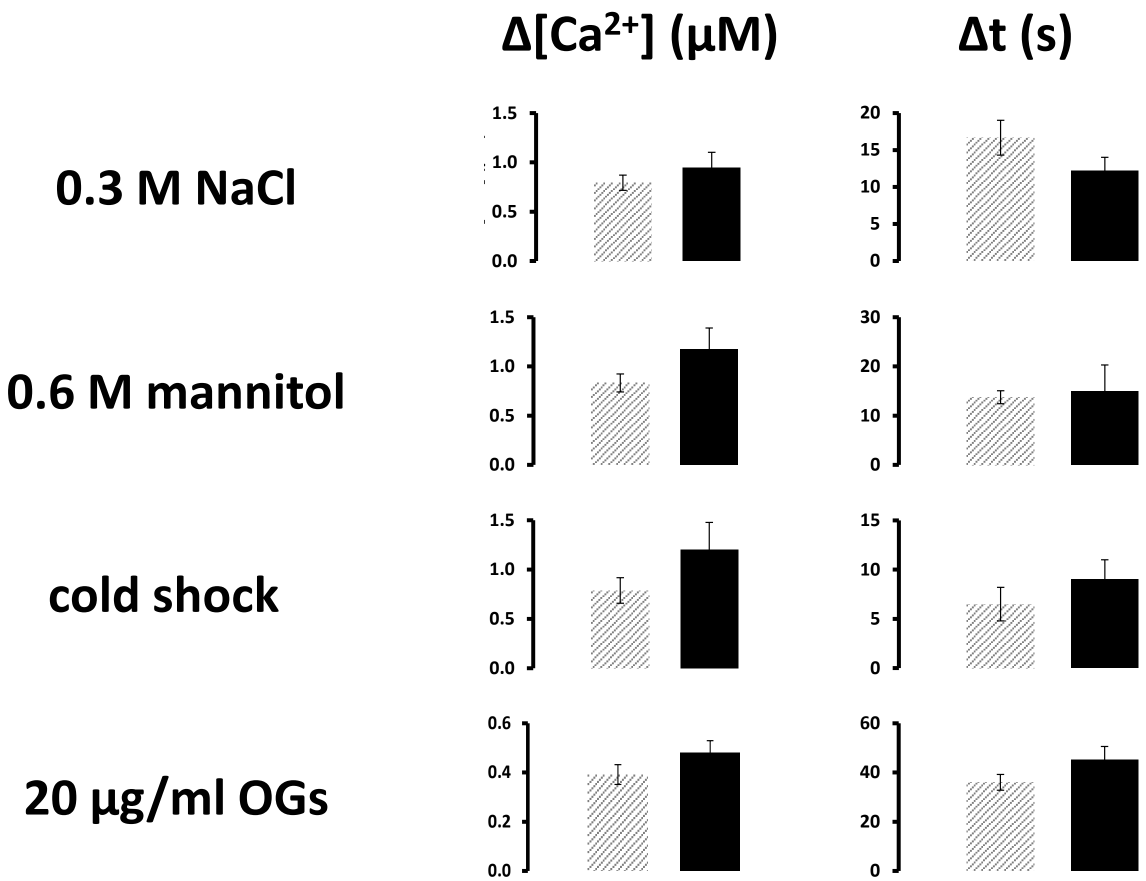

Supplementary Fig. S3. Analysis of stromal Ca^{2+} dynamics in terms of the difference between peak $[Ca^{2+}]$ and basal $[Ca^{2+}]$ ($\Delta[Ca^{2+}]$) and of the timing of the $[Ca^{2+}]$ peak after stimulus injection (Δt) in *A. thaliana* heterotrophic (grey columns) and dark-adapted autotrophic (black columns) cell suspension cultures in response to different environmental stimuli. No significant differences in either $\Delta[Ca^{2+}]$ or Δt of the Ca^{2+} peaks were observed between the two functional types of plastids.

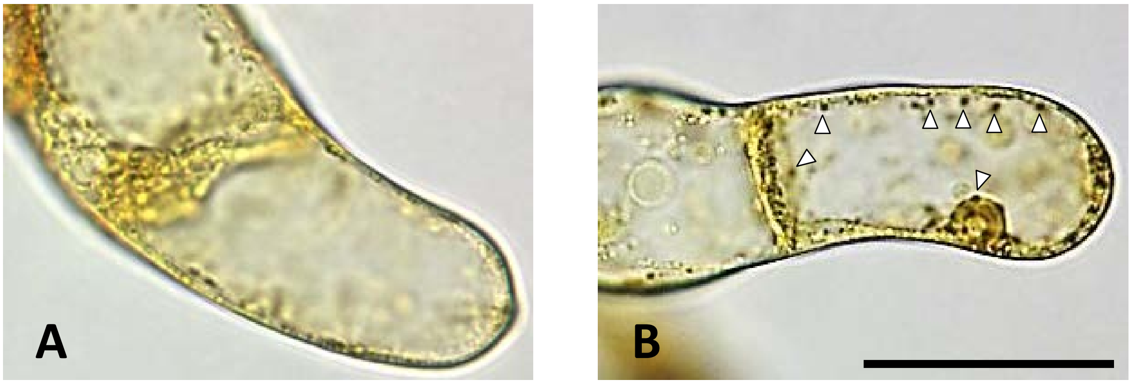

Supplementary Fig. S4. Reactivation of photosynthetic metabolism by 16 h-light exposure in *A. thaliana* autotrophic cell cultures. Lugol staining was used to detect transitory starch granules (white arrowheads) in chloroplasts, whose number was greatly reduced in suspension-cultured cells at the end of the 8 h dark phase (A) with respect to the end of the 16 h light phase (B). Bar, 50 µm.
